# Diffusion-Weighted Magnetic Resonance Application in Response Prediction before, during, and after Neoadjuvant Radiochemotherapy in Primary Rectal Cancer Carcinoma

**DOI:** 10.1155/2013/740195

**Published:** 2013-07-10

**Authors:** Daniela Musio, Francesca De Felice, Anna Lisa Magnante, Maria Ciolina, Carlo Nicola De Cecco, Marco Rengo, Adriano Redler, Andrea Laghi, Nicola Raffetto, Vincenzo Tombolini

**Affiliations:** ^1^Department of Radiotherapy, Policlinico Umberto I “Sapienza” University of Rome, Viale Regina Elena 326, 00161 Rome, Italy; ^2^Department of Radiological Sciences, Oncology and Pathology, Policlinico Umberto I “Sapienza” University of Rome, Polo Pontino, Corso della Repubblica 79, 04100 Latina, Italy; ^3^Department of Surgical Sciences, Policlinico Umberto I “Sapienza” University of Rome, Viale Regina Elena 326, 00161 Rome, Italy; ^4^Spencer-Lorillard Foundation, Viale Regina Elena 291, 00161 Rome, Italy

## Abstract

*Introduction*. Our interest was to monitor treatment response using ADC value to predict response of rectal tumour to preoperative radiochemotherapy. *Materials and Methods*. Twenty-two patients were treated with long course of radiochemotherapy, followed by surgery. Patients were examined by diffusion-weighted imaging MRI at three-time points (prior, during, and after radiochemotherapy) and were classified as responders and nonresponders. *Results*. A statistical significant correlation was found between preradiochemotherapy ADC values and during treatment ADC values, in responders (*F* = 21.50, *P* value <0.05). An increase in ADC value during treatment was predictive of at least a partial response. *Discussion*. Response of tumour to neoadjuvant therapy cannot be easily evaluated, and such capability might be of great importance in clinical practice, because the number of irradiated and operated patients may be superior to the number of who will really benefit from this multimodal treatment. A reliable prediction of the final clinical TN stage would allow radiotherapist to adapt multidisciplinary approach to a less invasive management, sparing surgical procedure in responder patients or even allowing an early surgery in nonresponders, which would significantly reduce radiochemotherapy related toxicity. *Conclusion*. Early evaluation of response during neoadjuvant radiochemotherapy treatment shows great promise to predict tumour response.

## 1. Introduction

Local control rates have improved in rectal cancer [1]. Nowadays, preoperative radiochemotherapy (RT-CHT) is standard treatment for patients with locally advanced rectal cancer, due to less acute toxicity, greater tumour response, and higher rates of sphincter preservation when compared with adjuvant therapy [2]. Improvement in pathological complete response (pCR) and feasibility of R0 resections on operative specimen have encouraged researchers to investigate a nonsurgical approach for clinical stage 0 disease following radiochemotherapy [3]. But an appropriate identification of complete clinical response is mandatory. Therefore, the chance to predict the response to neoadjuvant therapy before surgery would allow individualizing treatment. Diffusion-weighted magnetic resonance (DW-MRI) as an imaging biomarker has the potentiality for early evaluation of the response to RT-CHT treatment in a large section of cancer types, including head and neck tumours, pancreatic tumours, cervical tumours, and rectal cancer [4].

DW-MRI explores the random Brownian motion of water molecules in intracellular and extracellular space and, measuring water motion, reflects the biological changes in the tumour microenvironment, before and after treatment [5].

The aim of this study was to establish whether the use of DW-MRI as an imaging modality for response assessment during and after RT-CHT, in patients with locally advanced rectal cancer, can predict treatment outcomes, with the goal of improving RT-CHT technique or modified surgical approach.

## 2. Materials and Methods

### 2.1. Patient Selection

This was a prospective study and it was approved by our Institutional Review Board. Patients were enrolled after singing an informed consent. All patients had histologically proven rectal adenocarcinoma, clinically staged on pelvic magnetic resonance imaging (MRI) or endorectal ultrasound as T3-4 and/or with positive regional lymph-node, without any evidence of distant metastases by other imaging modalities. Patients were excluded from the study in case of synchronous tumours, cardiovascular disease, history of neurological or psychiatric disorders, or previous pelvic radiotherapy, contraindication to MRI examination. 

### 2.2. Treatment Plan

All patients were treated with a long course of RT-CHT. Radiation therapy was delivered with a 3D-conformational multiple field technique at a dose of 45 Gy (in 25 daily fractions of 1,8 Gy given in 5 weeks) to the whole pelvis, plus a 5,4–9 Gy (in 3–5 daily fractions of 1,8 Gy) to the tumour volume, with 6–15 MV energy photons. Chemotherapy consisted of 2-hour oxaliplatin infusion 50 mg/m^2^ on the first day of each week of radiotherapy and five daily continuous infusion of 5-FU 200 mg/m^2^/die. The choice of adding oxaliplatin to the standard schedule of 5-FU was dictated by our previous experience, in which the addition of oxaliplatin has resulted in a high rate of pathological complete response with acceptable toxicity [6].

Surgery was planned 7–9 weeks after the end of RT-CHT treatment. The type of surgery was chosen by the surgeon.

### 2.3. Magnetic Resonance Imaging

All patients were examined by MRI at three-time points: one week prior to RT-CHT (pre-RT-CHT MRI), at the beginning of the third week of RT-CHT (during RT-CHT MRI), and 6 weeks after the end of RT-CHT (post-RT-CHT MRI). All pre-, during, and post-RT-CHT MRI examinations were performed with a 3.0 T MR system (Discovery 750; GE Healthcare, Milwaukee, WI, USA) equipped with high-performance gradients (amplitude, 50 mT/m; slew rate 200 T/sec) using an 8-channel-phased array cardiac coil in the supine position. All patients underwent diffusion-weighted imaging (DWI) in addition to the conventional rectal MRI protocol. Axial DWI images were obtained using the single-shot echo-planar imaging technique. A spectral adiabatic inversion recovery fat saturation technique was used. Diffusion-encoding gradients were applied as a bipolar pair at 11 *b*-values of 0, 10, 20, 30, 50, 60, 100, 200, 600, 800, and 1000 sec/mm^2^, along the three orthogonal directions of the motion-probing gradients. The acquisition was separated in two blocks: the first block included *b*0, *b*10, *b*20, *b*30, *b*50, *b*60, and *b*100 while the second block included *b*200, *b*600, *b*800, and *b*1000; each block was acquired in free-breathing. The total acquisition time for DWI was approximately 5 minutes.

### 2.4. Imaging Analysis

Pre-, during, and post-RT-CHT MR images were analyzed independently by two gastrointestinal radiologists assessing on a dedicated workstation with advanced imaging analysis software. Tumour dimension and DWI parameter (apparent diffusion coefficient, ADC) were evaluated. Quantitative analysis of DWI was performed using the MATLAB code (Release 7.10.0, The Mathworks Inc., Natick, MA, USA). To calculate ADC, a region of interest (ROI) was drawn on the rectal cancer on b800 images (mean size 165 mm^2^; range, 100–230 mm^2^). Then ROI was transferred to all *b*-values images using an automated process. Mean signal intensities (SI) were obtained for each ROI with careful exclusion of the necrotic or cystic portions inside the tumour. 

The signal variation with increasing *b*-values was modeled by using the following biexponential function [7]: *S*_*b*_/*S*_0_ = (1 − *f*)*e* ^−*bD*^ + *fe* ^−*b*(*D*+D*)^, where *S*_*b*_ is the mean signal intensity with diffusion weighting *b*, *S*_0_ is the mean signal intensity for *b*-value of 0 s/mm^2^, *f* is the perfusion fraction, *D*, the diffusion coefficient (in mm^2^/s), and D* is the perfusion-related diffusion coefficient (in mm^2^/s).

### 2.5. Data Analysis

Pretreatment stage (cT cN) was compared with pathologic stage (ypT ypN). A partial response to treatment was defined as downstaging, or reduction of at least one level in T or N staging between the baseline RM exam and histopathological staging. Pathological complete response (pCR) was defined as the absence of any residual tumour cells detected in the operative specimen (ypT0 ypN0). No response to treatment was defined as stable or progressive disease.

### 2.6. Statistical Analysis

The mean ADC values pre-, during, and post-RT-CHT were compared with Fisher's test (*F*-test) both in all patients and between responders versus nonresponders. Following one-way analysis of variance (ANOVA), we compare the mean ADC of one group with the mean ADC of another at each time, using Fisher's least significant Difference test (LSD-test). A *P* value < 0.05 was considered statistically significant in the *F*-test and *P* value < 0.005 in the LSD-test to improve test power.

## 3. Results

Between February 2011 and May 2012, 22 consecutive patients, 12 men (mean age: 63,2 years; range: 50–71 years) and 10 women (mean age: 63,2 years; range: 47–81 years), were enrolled in the study. All patients underwent programmed RT-CHT neoadjuvant treatment. After RT-CHT, 15 (68.2%) and 7 (31.8%) of the 22 patients were classified as responders and nonresponders, respectively. Twenty patients underwent surgery, and pathological evaluation was performed in all of them. Fifteen patients showed a downstaging on the surgical evaluation. Of these, 9 patients (60%) had a pCR. 

Tumour ADC values of all 22 patients are reported in Table 1. The mean initial ADC value in patients with at least partial response was not significantly different than that in the nonresponders group. The evolution of the ADC values in the two groups was different. A statistical significant correlation was found between pre-RT-CHT ADC values and during treatment ADC values, in the responders (*F* = 21.50, *P* value < 0.05), so, this hypothesis was tested using least significant difference (LSD) method and result confirmed that the difference was significant (*T* = 4.26; *P* value < 0.005). No significant difference was observed among the during ADC and the posttreatment ADC values (*T* = 2.18; *P* value > 0.005), whereas, in the nonresponders group, *F*-test fails (*F* = 8.31, *P* value > 0.05).

## 4. Discussion

Multimodal treatment approach, combining radiotherapy, chemotherapy, and surgery, is the standard of care in locally advanced rectal cancer. RT-CHT gives the high chance of tumour downsizing and tumour downstaging, including pCR, as proven in randomised phase III trials, in which pCR rates range from 13 to 19.2% depending on preoperative RT-CHT regimens [8–10]. Moreover, several data demonstrated that a prolonged interval between neoadjuvant RT-CHT and surgery may still further improve pCR rates [11].

Pelvic magnetic resonance imaging (MRI) is the recommended diagnostic procedure for initial staging in rectal cancer [2], and it has recently been considered a noninvasive modality able to monitor treatment response, due to diffusion-weighted imaging (DWI) [12]. DW-MRI depends on the microscopic Brownian motion of water; the difference in water motion is quantified by the apparent diffusion coefficient (ADC), and it is inversely correlated to the tissue cellularity and the integrity of cell membranes. DW-MRI, therefore, provides microstructural information on tissue characterization and can estimate changes in cellularity of the tumour microenvironment, due to cellular death and vascular changes in response to treatment [5, 13]. So DW-MRI can help to discriminate posttreatment changes from residual active tumour. Usually, malignant tumour has lower ADC values, because of higher cellularity, tissue disorganization, and increased extracellular space tortuosity. After therapy, an increase in ADC values states a successful response to treatment, due to extracellular edema, and it is detectable before any change in tumour size [13, 14]. But water diffusion properties of tumour are not always so easy to interpret: in the same lesion can coexist different area with different cellular and vascular components, which, consequently, influence DWI properties [15]. So, the correlation ADC value-cellularity is not always preserved for adenocarcinomas and necrotic lesions, especially [13]. Lemaire et al. [16] evaluated induced rat mammary tumour response to chemotherapy using DW-MRI. Results showed that a high ADC value before treatment characterised tumours with high percentages of necrosis, predicting a worse chemosensitivity. Moreover, the ADC value varies dramatically during treatment, due to cell necrosis and lysis, and it makes difficult to understand the effects of treatment on DWI [15].

In rectal cancer DW-MRI has been used to predict tumour response to RT-CHT. Dzik-Jurasz et al. [17] compared baseline to posttreatment tumour ADC values in 14 patients with clinically advanced rectal adenocarcinoma. They noted poor response in pretreatment high tumour ADC, strengthening Lemaire's known relation between necrosis and worse response to treatment. Hein et al. [18] evaluated ADC in 16 patients with advanced rectal carcinoma submitted to neoadjuvant RT-CHT. After therapy, they reported a significantly lower ADC values in responders patients than in nonresponders. More recently Lambrecht et al. [19] came to similar correlation in 22 patients with adenocarcinoma of the rectum; they find that the initial ADC was significantly lower in patients with a pCR compared to patients with no pCR after RT-CHT.

Our interest was to monitor treatment response using ADC value to predict response of rectal tumour to RT-CHT. In this limited number of patients the initial ADC value shows no correlation with pCR. The initial ADC was not significantly different in responders versus nonresponders group. An ADC increase was observed in patients with at least a partial response to treatment. We have found that patients who respond to treatment show a significant rise in the ADC values after two weeks of therapy. An increase in the ADC value during treatment was predictive of at least a partial response. Our cohort group (*n* = 22) was, however, too small for a subgroup analysis. Study population increase would be necessary to provide predictive factors, such as ADC cut-off value. 

Four studies already reported on the use of DW-MRI during and after preoperative RT-CHT for rectal cancer [20–23]. The main message was similar. Changes in ADC values during course of RT-CHT correlated to biological changes in the tumour tissue, predicting tumour downstaging.

Kremser et al. [20] studied changes of ADC in 8 patients with primary rectal carcinoma. ADC values were determined and compared weekly during RT-CHT. Results showed a significant increase in ADC value during 1st week of treatment in responded group. Hein et al. [22] analyzed diffusion data of 9 patients undergoing preoperative RT-CHT for rectal cancer carcinoma. They demonstrated significant radiobiological changes during therapy by the detection of changes in water mobility.

Sun et al. [21] analysed, in a group of 37 patients with primary rectal carcinoma, whether changes in ADC values correlate with tumour histopathologic downstaging. ADC values were calculated at four-time point: before RT-CHT, at the end of the 1st week of therapy, at the end of the 2nd week of therapy, and before surgery. The study group was divided in downstaged patients and nondownstaged patients, based on pathological evaluation. At 1st week a significant ADC increase was recorded in the downstaged group. Barbaro et al. [23] evaluated prospectively a total of 62 patients with locally advanced nonmucinous rectal cancer. Patients underwent DW-MRI before, during (mean 12 days), and 6–8 weeks after RT-CHT. During treatment, tumour ADC was significantly greater in the responders group than in the nonresponders.

In clinical practice, according to Response Evaluation Criteria in Solid Tumors Group (RECIST), the objective tumour response to treatment is only characterized by the measurement of the lesion's longest diameter [24]. DW-MRI has been considered in the National Cancer Institute (NCI) consensus conference as integral part of oncologic imaging practice [13]. However, in case of clinical complete response (cCR) after preoperative RT-CHT, the standard treatment remains total mesorectal excision (TME) at the moment [2], because the benefit in using DW-MRI in the prediction of preoperative neoadjuvant therapy response in locally advanced rectal cancer is still debated [25].

Response of the tumour to neoadjuvant therapy cannot be easily evaluated, and such capability might be of great importance in clinical practice, because the number of irradiated and operated patients may be superior to the number of who will really benefit from this multimodal treatment. A reliable prediction of the final clinical T and N stages would allow the radiotherapist to adapt the multidisciplinary approach to a less invasive management, sparing the surgical procedure in responder patients or even allowing an early surgery in nonresponders, which would significantly reduce RT-CHT related toxicity. So an earlier and accurate prediction of the pathological tumour response during preoperative treatment could enable to guide modifications of treatment protocol. Further evidence is necessary to validate these observations.

## 5. Conclusions

DW-MRI provides important clinical information in management of patients with locally advanced rectal cancer. An early evaluation of response during neoadjuvant RT-CHT treatment shows great promise to predict tumour response. In our series, an increase in the ADC value during treatment was predictive of at least a partial response, with response being defined by pathological examination. DW-MRI should be tested in a large clinical study to demonstrate its accuracy to differentiate responders to nonresponders patients. 

## Figures and Tables

**Table 1:** Mean ADC value of responders and nonresponders at each time point.

Group	Time point			*F* value	*P* value
	Pre-RT-CHT	During RT-CHT	Post-RT-CHT		
Responders	0.87 ± 0.23	1.13 ± 0.26	1.28 ± 0.32	21.50	<0.05
Nonresponders	0.75 ± 0.14	1.03 ± 0.10	1.18 ± 0.38	8.31	>0.05

Data at time point are means ± standard deviations.
